# Spatio-temporal trends and risk factors affecting West Nile virus and related flavivirus exposure in Spanish wild ruminants

**DOI:** 10.1186/s12917-016-0876-4

**Published:** 2016-11-09

**Authors:** Ignacio García-Bocanegra, Jorge Paniagua, Ana V. Gutiérrez-Guzmán, Sylvie Lecollinet, Mariana Boadella, Antonio Arenas-Montes, David Cano-Terriza, Steeve Lowenski, Christian Gortázar, Ursula Höfle

**Affiliations:** 1 Departamento de Sanidad Animal, Facultad de Veterinaria, Universidad de Córdoba-Agrifood Excellence International Campus (ceiA3), Rabanales, 14071 Córdoba, Spain; 2 Instituto de Investigación en Recursos Cinegéticos IREC, (CSIC-UCLM-JCCM), Ciudad Real, Spain; 3 ANSES, Laboratoire de Santé Animale de Maisons-Alfort, UMR 1161 Virologie, INRA, ANSES, ENVA, Maisons-Alfort, F-94703 France; 4 Sabiotec, Camino de Moledores s.n., Ed. Polivalente UCLM, 13005 Ciudad Real, Spain

**Keywords:** West Nile virus, Usutu virus, Meaban virus, Red deer *Cervus elaphus*, Risk factors, Wild ruminants, Spain

## Abstract

**Background:**

During the last decade, the spread of many flaviviruses (Genus *Flavivirus*) has been reported, representing an emerging threat for both animal and human health. To further study utility of wild ruminant samples in West Nile virus (WNV) surveillance, we assessed spatio–temporal trends and factors associated with WNV and cross-reacting flaviviruses exposure, particularly Usutu virus (USUV) and Meaban virus (MBV), in wild ruminants in Spain. Serum samples from 4693 wild ruminants, including 3073 free-living red deer (*Cervus elaphus*), 201 fallow deer (*Dama dama*), 125 mouflon (*Ovis aries musimon*), 32 roe deer (*Capreolus capreolus*) and 1262 farmed red deer collected in 2003–2014, were screened for WNV and antigenically-related flavivirus antibodies using a blocking ELISA (bELISA). Positive samples were tested for neutralizing antibodies against WNV, USUV and MBV by virus micro-neutralization tests.

**Results:**

Mean flavivirus seroprevalence according to bELISA was 3.4 ± 0.5 % in red deer, 1.0 ± 1.4 % in fallow deer, 2.4 ± 2.7 % in mouflon and 0 % in roe deer. A multivariate logistic regression model revealed as main risk factors for seropositivity in red deer; year (2011), the specific south-coastal bioregion (bioregion 5) and presence of wetlands. Red deer had neutralizing antibodies against WNV, USUV and MBV.

**Conclusions:**

The results indicate endemic circulation of WNV, USUV and MBV in Spanish red deer, even in areas without known flavivirus outbreaks. WNV antibodies detected in a free-living red deer yearling sampled in 2010, confirmed circulation this year. Co-circulation of WNV and USUV was detected in bioregions 3 and 5, and of WNV and MBV in bioregion 3. Sampling of hunted and farmed wild ruminants, specifically of red deer yearlings, could be a complementary way to national surveillance programs to monitor the activity of emerging flaviviruses.

## Background

The distribution of vector-borne flaviviruses (family *Flaviviridae*) in the world has substantially increased over the last decades. During this period, many flavivirus infections have become a major public health concern due to continuous and growing reporting of outbreaks in humans [1]. Flaviviruses are mainly transmitted within an enzootic cycle involving ornithophilic mosquitoes or ticks as competent vectors, as well as wild birds as the main amplifying hosts in the wild. Most mammalian species including humans are considered dead-end or incidental hosts, because they can get infected but are not thought to be able to transmit the viruses.

During the last few years, six flaviviruses, including West Nile virus (WNV), Usutu virus (USUV), tick-borne encephalitis virus (TBEV), Bagaza virus (BAGV), Meaban virus (MBV) and louping-ill virus (LIV), have been detected in Europe [2, 3]. Five of them have circulated in Spain in the last decade. WNV exposure has been documented in mosquitoes [4], wild birds [5, 6] and different mammalian species, including humans [7], horses [8], dromedary camels (*Camelus dromedarios*) [9], wild boar (*Sus scrofa*) and Iberian pigs and red foxes (*Vulpes vulpes*) [10]. Clinical disease and mortality associated with WNV infection has also been detected in wild birds, horses and humans in this country [11, 12]. USUV has been detected in mosquitoes [4, 13] and in both migratory and resident birds in Spain [14, 15]. Mortality associated with Louping-ill virus (LIV) infection was also detected in sheep and goats in northern Spain [16] and suspected in chamois (*Rupicapra pyrenaica*) [17]. An unusually high mortality due to Bagaza virus (BAGV) infection was also confirmed in free-living game birds in south-western Spain in 2010 [18]. Finally, Meaban virus (MBV) has been found in both yellow-legged gulls (*Larus michaelis*) and ticks (*Ornithodoros maritimus*) in north-eastern Spain [3].

Wild and domestic artiodactyls can be useful sentinel species for monitoring flavivirus activity [19–21]. Antibodies against St. Louis encephalitis virus (SLEV) and WNV have been found in white-tailed deer (*Odocoileus virginianus*) from the United States [22, 23], and against TBEV and WNV in different game species from the Czech Republic [24]. In addition, fatal cases of WN fever were reported in white-tailed deer and reindeer (*Rangifer tarandus*) in North America [25, 26]. Serosurveillance on WNV and related flaviviruses has also been performed in wild and domestic Spanish animals such as wild boar, red deer (*Cervus elaphus*), Iberian pigs, red foxes, and others [8, 10]. Accordingly, 0.2 % yearling red deer from south-western Spain had antibodies against WNV or cross-reacting flaviviruses [27].

As red deer is an important game species in Spain [28] and is also frequently farm raised, it could be an easily accessible, cost-effective species to use as a complementary tool to the national surveillance programs to monitor the activity of mosquito-borne flaviviruses [27]. While the analysis of samples from yearlings is useful for continuous surveillance and early warning systems, the (retrospective) analysis of serum samples of individuals of all age classes is a useful tool to explore temporal and spatial trends of flavivirus activity in a given region [23].

The aim of this study was to monitor seroprevalence of WNV and antigenically-related mosquito and tick-borne flaviviruses, particularly USUV and MBV, in wild ruminants in Spain, and, using red deer as the most distributed, abundant and readily available species, to assess the spatial–temporal trends and risk factors associated with the exposure to these flaviviruses in this species.

## Methods

### Study area

Samples of 4693 wild ruminants, including 3073 free-living and 1262 farmed red deer, 201 fallow deer (*Dama dama*), 125 mouflon (*Ovis aries musimon*) and 32 roe deer (*Capreolus capreolus*) were collected between 2003 and 2014 from hunting estates across Spain. Red deer were sampled in 130 hunting areas located throughout the five geographical bioregions of Spain previously defined for the national wildlife disease monitoring program [29, 30]: (1) Atlantic coast, characterized by a wet, temperate climate and abundant rainy seasons (32 sampling areas); (2) cereal plains, in which agriculture with cereal crops are dominant (7 sampling areas); (3) Continental Mediterranean ecosystem, with cold winters, hot dry summers, and rainy seasons in autumn and spring (70 sampling areas); (4) Interior mountains, consisting of small mountain chains with a continental Mediterranean climate (9 sampling areas); (5) and south coast, with a humid coastal Mediterranean climate, warm humid winters and hot dry summers (12 sampling areas). Samples of roe deer, fallow deer, and mouflon, were obtained only from bioregions 3 and 5 (south-central Spain).

### Sampling

Blood samples were taken from the thoracic cavity of freshly killed hunted animals or during health inspection in the slaughterhouse. Samples of farmed red deer were obtained by puncture of the jugular vein using a sterile collection system during health inspections. All samples of free-living ruminants were grouped by season and habitat type (sites associated with large permanent wetlands i.e., rivers, lagoons or lakes, and areas without wetlands or large water bodies). Samples were classified by age according to dentition patterns (yearlings, sub-adults, adults) [31]. The animals sampled were classified according to gender and status (farmed or free-living). When kidney samples were available from red deer, the right kidney fat index (KFI) was obtained as an indicator of body condition [32]. Upon arrival at the laboratory, blood samples were centrifuged for 15 min at 1,800X *g* for serum separation and serum was stored at −20 ° C until analysis.

### Laboratory analysis

All ruminants were tested for antibodies against an epitope of the WNV pre-membrane-envelope (prM-E) protein shared with other viruses of the Japanese encephalitis serocomplex. A commercially available blocking enzyme-linked immunosorbent assay (bELISA 10.WNV.K3 INGEZIM West Nile COMPAC®, Ingenasa, Madrid, Spain) was used in accordance with the manufacturer’s recommendations. The bELISA was used as a serological screening tool and bELISA-positive and doubtful sera were confirmed by micro virus neutralization test (VNT) for the detection of specific neutralizing antibodies against WNV (Is98 strain, lineage 1) and USUV (It12 strain). Additionally, given the possibility of cross-reaction with antigenically-related flaviviruses not included within the Japanese encephalitis serocomplex such as Meaban virus (MBV), bELISA-positive and doubtful samples were also tested by VNT against this flavivirus (MBV; Brest ART707 strain). VNTs were performed as previously described [3, 33]. Samples that showed neutralization and absence of cytopathic effect at dilutions ≥ 10 for WNV and USUV and ≥ 20 for MBV were considered positive. Interpretation of results was based on comparison of VNT titers obtained in parallel against the three flaviviruses. The neutralizing immune response observed was considered specific when VNT titers for a given virus was ≥ 4-fold higher than titers obtained for the other viruses. Samples showing VNT titers differences ≤ 2-fold between the viruses examined were considered positive for flaviviruses but not conclusive for any specific virus.

### Statistical analysis

The prevalence of antibodies against antigenically-related flaviviruses was estimated from the ratio of positives to the total number of samples, with the exact binomial confidence intervals of 95 %. Differences between species were analyzed using a Pearson’s Chi-square test and a Fisher’s exact test (when observations/category were <6). Due to the absence of seropositivity in roe deer and to the limited number of seropositive fallow deer and mouflon, the associated risk factors were only analyzed for red deer, the most widely distributed and abundant wild ruminant species in Spain. A Chi-square or Fisher’s exact test were used to test the relevance of the explanatory variables (age class, gender, season, year, status, bioregion and wetland area) to the risk of an red deer being exposed to flaviviruses. Covariates correlated with a *P*-value < 0.20 in the bivariate analysis were included for further analysis. Biologically plausible confounding factors were assessed using Mantel-Haenszel analysis and confounding was considered to be potentially significant if odd ratios (ORs) shifted appreciably. Variables that altered the coefficients for the independent variables of interest by 30 % or more were removed from the model and were classified as confounding factors. Finally, a multiple logistic regression analysis [34] was performed including risk factors potentially associated with related flavivirus exposure (likelihood-ratio Wald’s test, *P* < 0.05). The goodness of fit was assessed using the Hosmer–Lemeshow goodness-of-fit test. SPSS 22.0 software (IBM Corp., Armonk, NY, USA) was used for statistical analyses.

## Results

A total of 153 out of the 4693 (3.3 %, CI_95%_: 2.7–3.8 %) wild ruminants were seropostive against antigenically-related flaviviruses using bELISA. A mean seroprevalence of 3.4 ± 0.5 % (148/4335) was obtained in red deer, 1.0 ± 1.4 % (2/201) in fallow deer, and 2.4 ± 2.7 % (3/125) in mouflon. No seropositivity was detected in the 32 roe deer tested. Statistically significant differences in seroprevalence among species were not observed.

Five (year, bioregion, wetland area, season and status) out of seven variables were selected (*P* < 0.20) from the bivariate analysis (Table 1). At least one seropositive red deer was detected in every year analyzed, with a significantly higher seroprevalence detected in 2011 (17.6 %; 44/250; *P* < 0.001). Forty-one out of the 130 (31.5 %) sampling areas presented at least one seropositive animal. The seropositivity by sampling areas was 3.1 % (1/32) in bioregion 1, 42.9 % (3/7) in bioregion 2, 44.3 % (31/70) in bioregion 3, 11.1 % (1/9) in bioregion 4 and 41.7 % (5/12) in bioregion 5. Even though seropositivity was detected in all bioregions, considering farmed and free-living red deer together, a significantly higher seroprevalence was found in bioregion 5 (7.9 %; 79/998) compared to the other bioregions (*P* < 0.001), and in bioregion 3 (2.8 %; 61/2164) compared to bioregion 1 (0.2 %; 1/437) (*P* < 0.001). Significantly higher seropositivity was also detected in bioregion 5 (3.0 %; 8/269) (*P* = 0.003), bioregion 4 (3.0 %; 4/134) (*P* = 0.012) and bioregion 3(2.9 %; 59/2017) (*P* < 0.001) compared to bioregion 1 (0.2 %; 1/437) when seroprevalence was compared only in free-living red deer. A significantly higher seroprevalence was obtained in summer (5.8 %; 29/503) as compared to autumn (3.0 %; 52/1754) (*P* = 0.003) and winter (3.4 %; 57/1701) (*P* = 0.012). Seroprevalence was significantly higher in farmed 5.9 % (74/1262) than in free-living 2.4 ± 0.5 % (74/3073) red deer (*P* < 0.001). Seropositivity was confirmed in three out of the five red deer farms tested, with the mean seroprevalence ranging from 0.4 % (1/243) to 9.7 % (71/729). In the red deer farm with the highest seropositivity, a significantly higher mean seroprevalence was found in autumn (18.5 %; 20/108) compared to spring (4.6 %; 6/131; *P* = 0.002) and summer (8.4 %; 26/309; *P* = 0.012), but not in winter (10.5 %; 19/181; *P* = 0.113). The final multivariate logistic regression model showed that the main risk factors associated with exposure to related flaviviruses in red deer were: year (2011), bioregion (5) and wetland areas (presence) (Table 2).

**Table 1:** Seroprevalence to WNV and antigenically-related flaviviruses in wild ruminants in Spain

Variable	Categories	No. examined^a/^positive (%)	*P*-value
Species	Red deer	4335/148 (3.4)	0.174
	Fallow deer	201/2 (1.0)	
	Mouflon	125/3 (2.4)	
	Roe deer	32/0 (0.0)	
Age class	Juveniles	56/1 (1.8)	0.628
	Sub-adults	221/8 (3.6)	
	Adults	607/26 (4.3)	
Sex	Females	949/24 (2.5)	0.217
	Males	893/29 (3.2)	
Bioregion	1	437/1 (0.2)	<0.001
	2	506/3 (0.6)	
	3	2164/61 (2.8)	
	4	230/4 (1.7)	
	5	998/79 (7.9)	
Year	2003	502/9 (1.8)	<0.001
	2004	179/1 (0.6)	
	2005	338/5 (1.5)	
	2006	446/12(2.7)	
	2007	664/13 (2.0)	
	2008	551/15 (2.7)	
	2009	555/21 (3.8)	
	2010	576/20 (3.5)	
	2011	250/44 (17.6)	
	2012	74/1 (1.4)	
	2013	140/5 (3.6)	
	2014	60/2 (3.3)	
Season	Autumn	1754/52 (3.0)	0.017
	Spring	377/10 (2.7)	
	Summer	503/29 (5.8)	
	Winter	1701/57 (3.4)	
Wetland area	Presence	1634/109 (6.7)	<0.001
	Absence	2701/39 (1.4)	
Status	Free-living	3073/74 (2.4)	<0.001
	Farmed	1262/74 (5.9)	

^a^Missing values excluded

**Table 2:** Logistic regression model of potential risk factors associated with seroprevalence to WNV and antigenically-related flaviviruses in red deer in Spain

Variable	Categories	*B*	*P*-value	OR	95 % CI	
Year	2003	_a_	_a_	_a_	_a_	_a_
	2004	−1.174	0.271	0.309	0.038	2.496
	2005	−0.028	0.962	0.972	0.313	3.020
	2006	0.294	0.525	1.342	0.542	3.322
	2007	0.223	0.627	1.250	0.509	3.072
	2008	0.430	0.332	1.538	0.645	3.669
	2009	0.509	0.219	1.663	0.739	3.743
	2010	0.386	0.355	1.471	0.649	3.335
	2011	1.991	<0.001	7.320	3.404	15.739
	2012	−0.741	0.487	0.477	0.059	3.851
	2013	0.077	0.892	1.081	0.353	3.306
	2014	0.608	0.447	1.837	0.383	8.822
Bioregion	1	_a_	_a_	_a_	_a_	_a_
	2	0.602	0.605	1.826	0.186	17.946
	3	1.913	0.060	6.777	0.920	49.903
	4	1.784	0.115	5.954	0.648	54.709
	5	2.192	0.034	8.957	1.180	67.990
Wetland area	Absence	_a_	_a_	_a_	_a_	_a_
	Presence	1.097	<0.001	2.995	1.888	4.751

^a^Reference category

**Fig. 1:**
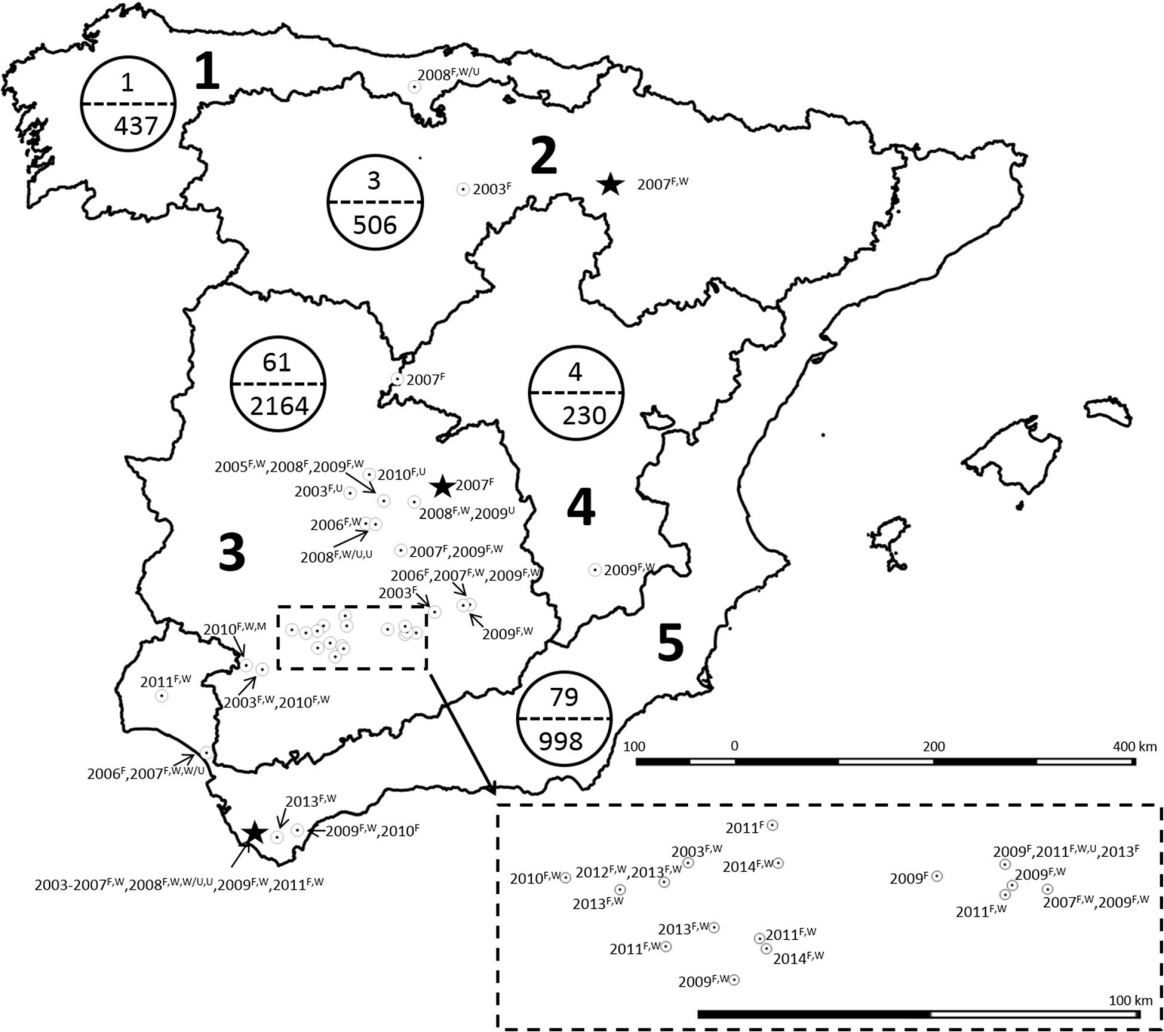
Detection of antibodies to WNV and antigenically-related flaviviruses in red deer in the five different bioregions of Spain (large circles positives/n tested) and spatio-temporal distribution of exposure to flaviviruses, WNV, USUV and Meaban virus. *Open circles* represent hunting estates (only the 41 positive hunting estates are included), *black stars* the red-deer farms. Years in which antibody positive animals were detected are listed with superscript letters that list the pathogens against which antibodies were present: F = Flavivirus ELISA positive, W = WNV, W/U = WNV/USUV, U = USUV, M = MBV

Testing by VNT was possible in 140 out of the 153 bELISA seropositive wild ruminants, while 13 samples (11 from red deer and 2 from fallow deer) could not be analyzed by VNT due to serum cytotoxicity. Of these, 25 (22 from red deer and 3 from mouflon) showed negative results against the three flaviviruses tested using VNT. Specific antibodies against WNV, USUV and MBV were confirmed in 103 (69.7 %; bioregions 2, 3, 4 and 5), 4 (2.7 %; bioregions 1, 3 and 5) and 2 (1.4 %; bioregion 3) red deer, respectively (Table 3, Fig. 1). Six red deer (five from bioregion 3 and one from bioregion 5) showed ≤ 2-fold VNT titer differences between WNV and USUV and were therefore considered positive for other related flaviviruses but not conclusive for any of these viruses. Taking into account possible co-infections, the overall antibody prevalence ranged between 2.2 and 2.4 % for WNV, 0.1 and 0.2 % for USUV and 0.04 % for MBV. Specific WNV, USUV and MBV antibodies were confirmed in 31, four and one sampling areas, respectively. WNV and USUV and WNV and MBV co-circulation (different individuals from the same sampling area with specific antibodies against WNV, USUV or MBV) were detected in two (bioregions 3 and 5) and one (bioregion 3) sampling areas, respectively (Fig. 1).

**Table 3:** Virus neutralization test (VNT) titers obtained in parallel against West Nile virus (WNV), Usutu virus (USUV) and Meaban virus (MBV) from 115 sera positive by bELISA

VNT Titers	WNV	USUV	MBV	WNV and/or USUV
10	28	3		4 (WNV); 3 (USUV)
20	31		2	1 (WNV); 2 (USUV)
40	18	1		
80	12			
160	8			
320	3			1 (USUV)
≥640	3			1 (WNV)

## Discussion

The overall prevalence of antibodies against WNV and antigenically-related flaviviruses detected in wild ruminants (3.3 %) in the present study was consistent with that recently observed in captive zoo artiodactyls in Spain [15]. Similar seroprevalences were also found in white-tailed deer in the United States [22, 23]. In contrast, a limited number of wild ruminants from south-western Spain tested during surveillance of WNV outbreaks were negative for flavivirus antibodies using the same bELISA [8]. In our study, no antibodies were detected in roe deer, potentially due to the small sample size, while a similar study in the Czech Republic reported flavivirus exposure in roe deer, mouflons, fallow deer and red deer [24]. Remarkably, wild ruminants and specifically red deer showed lower seroprevalence to WNV and related flaviviruses than other artiodactyl species such as wild boar from the same regions and periods [10, 27]. In North America, *Culex* family mosquitoes (flavivirus vector) have shown a preference of feeding on deer [35]. Similarly, *Culex* species sampled in Canary Islands (Spain) frequently fed on ruminant species [36], while a study in wetlands of bioregion 5 did show feeding on mammals in addition to birds and reptiles but without identifying the mammal hosts beyond humans and horses [37]. Thus, our results would suggest a reduced susceptibility of red deer to flaviviruses of the encephalitis antigenic complex and other cross reacting flaviviruses.

Multivariate logistic regression identified the year 2011, bioregion 5 and the presence of wetland areas as individual risk factors for antigenically-related flavivirus exposure in red deer. The seroprevalence data obtained indicates widespread but not homogeneous distribution of these flaviviruses in wild ruminants in Spain. Consistently with other studies, seroprevalence of flaviviruses in both farmed and free-living wild ruminants, mainly WNV, was higher in southern Spain, namely in bioregions 3, 4 and 5 [10, 38, 39]. In fact, bioregion 5 includes the provinces with the highest number of WNV outbreaks reported in horses and humans and the area where BAGV infection caused high mortality in wild game birds in 2010 [18, 40]. The results indicate that southern regions present the highest risk of flavivirus circulation in Spain. Even though a study conducted in 1980 described the presence of antibodies against flaviviruses in rodents in northern Spain [41], the extremely low prevalence (0.2 %) of flavivirus antibodies in north Spain (bioregion 1), which has a colder mean temperature [42], suggests lower activity of flaviviruses in this area.

Animals sampled in wetland areas were three times more likely to be exposed to related flaviviruses, which may be due to the larger populations and greater diversity of competent vectors. In addition, in late summer, the number of wetlands and waterholes in southern Spain becomes greatly reduced, aggregating animals in these hotspots [43], which may also act as breeding areas for mosquito species. Our results are consistent with those previously reported in France [44], and indicate that wetland areas may help to target risk-based surveillance in Spain.

Seropositivity of at least one animal in all sample years supports a more-or-less continuous circulation of antigenically-related flaviviruses in the study period. However, a significantly higher seroprevalence was found in 2011, 1 year after the highest number of WNV and Bagaza outbreaks were reported in Spain [18, 40]. In 2011, seroprevalence was detected in adult and sub-adult deer but not in yearling animals suggesting exposure to related flaviviruses of the wild ruminant populations at the end of 2010 and persistence of antibodies in infected animals. Accordingly, a seropositive yearling free-living red deer sampled in 2010 was positive to WNV by VNT, confirming WNV circulation this year. Similar results were obtained in juvenile red deer from a farm in the 2010 outbreak area [27]. Our results are in agreement with previous studies in which an increase in WNV seroprevalence was observed in red deer and white-tailed deer 2 years after the first outbreak occurred in the area [23, 27].

Intrinsic host factors such as age can influence exposure and susceptibility to flavivirus infection. In the present study, seroprevalence increased with age but no significant differences among age classes were observed. The results may be explained by the limited number of samples from yearlings analyzed, due to the fact that yearlings are not usually hunted. A low seroprevalence in yearling red deer from Spain was also previously reported by [27]. Moreover, lower seroprevalences were detected in yearling while-tailed deer but no association with age was observed [22, 23]. Our findings are also consistent with the increase in WNV and related flavivirus seroprevalence with age found in wild boar [10]. Although flavivirus antibody persistence in artiodactyl species is unknown, a longer time span of possible exposure and antibody persistence could be associated with the higher seroprevalence found in adult individuals. Further research with larger sample sizes for the different age classes is needed to have a better understanding about the association between age and flavivirus infection.

Significantly higher seroprevalence to antigenically-related flavivirus was found in farmed red deer as compared to free-living ones, although this variable was not retained in the multivariate analysis. In contrast, free-living red deer sampled in open and fenced sampling areas did not differ in seroprevalence. Our results indicate that samples from farmed red deer, which are accessible during all year, may be a valuable surveillance tool. Variations in the seroprevalence among seasons were also detected in the bivariate analysis, with significantly higher seroprevalence in summer. Moreover, significantly higher mean seroprevalence was found in autumn in farmed red deer sampled in bioregion 5. Most of the WNV outbreaks in horses in Spain have been reported in late summer and autumn [40], confirming the higher risk of flavivirus infection during these seasons. In addition, the higher seropositivity observed in autumn in farmed deer is also in agreement with what has been reported in wild birds in Spain [5, 6].

To the authors’ knowledge, this is the first report on WNV, USUV and MBV antibodies in red deer, which confirms local circulation of these flaviviruses in Spain. Twenty two sera were positive by bELISA but negative to USUV, WNV and MBV in VNT. The results could be due to the circulation of other related flaviviruses [45]. Mortality due to BAGV and LIV infections have recently been detected in wild game birds and ruminant species respectively in Spain [16, 18]. Further studies are needed to determine the possible impact of these flaviviruses on wild ruminant populations. Unfortunately, VNT against BAGV and LIV could not be carried out in the present study. Six red deer presented positive results to WNV and USUV by VNT with titer differences ≤ 2-fold. This could be due to VNT cross reactions, however co-infections cannot be ruled out. In fact, both WNV and USUV as well as WNV and MBV co-circulation were confirmed (in two and one sampling areas, respectively). These results are of interest because a previous infection with a closely related flavivirus might confer cross-reactive immunity in the animal host, reducing the amplification of new strains with higher virulence introduced into the same region [46]. In fact, co-circulation of different flaviviruses, and the induction of cross-protective antibodies, has been suggested as an explanation for the high rate of seropositivity to WNV in birds and the limited number of cases reported in humans [46]. In Spain, both WNV lineage 1 and the putative new lineage, as well as BAGV, USUV and MB virus have been confirmed [47]. In contrast, the understanding of flavivirus co-infections both in host and vector species is still very limited.

Specific antibodies against WNV were confirmed in four out of the five bioregions. The higher WNV seroprevalence in bioregion 3 and 5 is in accordance with WNV distribution reported in horses in Spain [40]. Interestingly, WNV antibodies were found in animals in areas where WNV outbreaks have not been reported to date. Our results constitute the first report of WNV circulation in bioregion 4, where habitat conditions do not favour WNV activity. Yearly re-emergence of WNV in Mediterranean countries is thought to be due to WNV overwintering and becoming endemic in local bird populations [48, 49]. The presence of animals seropositive to WNV throughout the sampling period (2003–2014) suggest an endemic circulation of WNV in southern Spain. In fact, after WNV was first reported in horses from southern Spain in 2010, outbreaks in this species have been reported every year [40].

During the last decade, USUV has rapidly expanded in different European countries, and has been detected in mosquitoes and vertebrate species [50]. Even though human infections are believed to be clinically mild or asymptomatic, neuroinvasive illness associated with USUV infection has been reported in Italy and Croatia [51, 52]. In our study, USUV infection was confirmed in red deer from bioregions 1, 3 and 5 in 2007 and 2008. The seroprevalence found in this species, as well as the USUV infections previously reported in mosquitoes, resident and migratory wild birds [4, 14, 15, 53], indicate circulation of this emerging flavivirus during the last decade in Spain. Further epidemiological and molecular research is required to determine the role of wild ruminants in the epidemiology of USUV and to determine USUV strains and lineages circulating in Spain.

Infection with MBV or a Meaban-like virus has been recently detected in yellow-legged and herring (*Larus argentatus*) gulls from different breeding colonies in France and Spain [3], and in a great frigatebird (*Fregata minor*) and a sooty tern (*Onychoprion fuscatus*) in the Western Indian Ocean [54], although the zoonotic potential of this emerging flavivirus is still unknown. Two adult free-living red deer sampled in Seville province (bioregion 3) in 2010 showed antibodies against this flavivirus, which indicates that MBV is also circulating among wild ruminants in Spain. Interestingly, seropositivity against MBV has been detected precisely in the same province and year in a recent study carried out in waterfowl used as decoys in Spain (unpublished data), which suggests limited spread of this flavivirus in the study area. This result is consistent with a previous report of MBV infection in only two specific locations within the Mediterranean Basin [3].

## Conclusions

The results obtained indicate local circulation of antigenically-related flaviviruses in wild ruminants in Spain. The significance of WNV, USUV and MBV infection in this species is unknown at this time and further research on aspects such as the genetic diversity, infection rates and competent vectors of these emerging flaviviruses is needed. Surveillance of hunted wild ruminants, and especially on farmed red deer, may be a useful, cost-effective, rapid and complementary tool to the national surveillance programs to monitor the activity of mosquito and tick-borne flaviviruses in Europe.

## Abbreviations

BAGV: Bagaza virus
ELISA: Enzyme-linked immunosorbent assay
KFI: Kidney fat index
LIV: Louping-ill virus
MBV: Meaban virus
ORs: Odd ratios
SLEV: Saint Louis encephalitis virus
TBEV: Tick borne encephalitis virus
USUV: Usutu virus
VNT: Virus neutralization test
WNV: West Nile virus
